# Von Willebrand Factor Antigen Predicts Response to Double Dose of Aspirin and Clopidogrel by PFA-100 in Patients Undergoing Primary Angioplasty for St Elevation Myocardial Infarction

**DOI:** 10.1155/2013/313492

**Published:** 2013-12-19

**Authors:** Jacopo Gianetti, Maria Serena Parri, Francesca Della Pina, Federica Marchi, Endrin Koni, Alberto De Caterina, Stefano Maffei, Sergio Berti

**Affiliations:** Operative Unit of Cardiology, Fondazione Gabriele Monasterio, Ospedale del Cuore “G. Pasquinucci”, Via Aurelia Sud, 54100 Massa, Italy

## Abstract

Von Willebrand factor (VWF) is an emerging risk factor in acute coronary syndromes. Platelet Function Analyzer (PFA-100) with Collagen/Epinephrine (CEPI) is sensitive to functional alterations of VWF and also identifies patients with high on-treatment platelet reactivity (HPR). The objective of this study was to verify the effect of double dose (DD) of aspirin and clopidogrel on HPR detected by PFA-100 and its relation to VWF and to its regulatory metalloprotease ADAMTS-13. Between 2009 and 2011 we enrolled 116 consecutive patients with ST elevation myocardial infarction undergoing primary PCI with HPR at day 5 after PCI. Patients recruited were then randomized between a standard dose (SD, n = 58) or DD of aspirin and clopidogrel (DD, n = 58), maintained for 6 months follow-up. Blood samples for PFA-100, light transmittance aggregometry, and VWF/ADAMTS-13 analysis were collected after 5, 30, and 180 days (Times 0, 1, and 2). At Times 1 and 2 we observed a significantly higher CEPI closure times (CT) in DD as compared to SD (P < 0.001). Delta of CEPI-CT (*T*1 − *T*0) was significantly related to VWF (P < 0.001) and inversely related to ADAMTS-13 (0.01). Responders had a significantly higher level of VWF at *T*0. Finally, in a multivariate model analysis, VWF and ADAMTS-13 in resulted significant predictors of CEPI-CT response (P = 0.02). HRP detected by PFA-100 in acute myocardial infarction is reversible by DD of aspirin and clopidogrel; the response is predicted by basal levels of VWF and ADAMTS-13. PFA-100 may be a useful tool to risk stratification in acute coronary syndromes given its sensitivity to VWF.

## 1. Introduction

In the pathophysiology of arterial thrombosis VWF is a critical mediator in the very early phases of platelets adhesion to the subendothelial under high shear stress conditions [1–3]. VWF levels and activities are contraregulated by ADAMTS-13, a metalloprotease, a severe defective activity of which, genetically determined, provokes a thrombotic condition known as thrombocytopenic thrombotic purpura syndrome [4]. Because of these premises, it is not surprising that VWF is a risk factor in acute vascular syndromes and an emerging target of novel antithrombotic therapeutics [5].

Platelet Function Analyzer (PFA-100) with Collagen/Epinephrine (CEPI) cartridges is sensitive to functional alterations of VWF and also identifies patients with high residual platelet reactivity (HPR) on dual antiplatelet therapy (DAPT) [6–8]. PFA-100 is a COX-1 independent assay and the relatively high prevalence of HPR detected by this method, not always confirmed by light transmission aggregation with arachidonic acid (LTA-AA), is explained by this characteristic [9].

Whether the risk of high VWF levels is indirectly recognizable by PFA-100 and correctable by a tailoring strategy based on PFA-100 results is presently unknown.

Thus, the objective of this study was to verify the effect of a double dose of DAPT on HPR detected by PFA-100 and its relation to VWF and to its regulatory metalloprotease ADAMTS-13.

## 2. Materials and Methods

### 2.1. Study Design

This study was a randomized, open-label, active comparator-controlled study of a standard versus tailored DAPT in patients with ST elevation myocardial infarction undergoing primary percutaneous coronary intervention (PCI).

The study protocol was approved by the local Ethical Committee and informed consent was obtained from all subjects (*n* = 116) who entered the study between July 2009 and December 2011.

**Figure 1:**
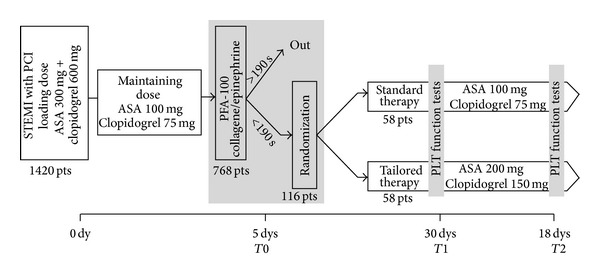
Study design.

We excluded patients older than 80s, rescue PCI, angiographic documentation of left main branch or three vessels disease requiring urgent surgical revascularization, acute renal failure treated by continuous venous-venous hemodialysis, or respiratory insufficiency requiring mechanical ventilation. Preoperative use of oral anticoagulants on a permanent basis or aspirin intolerance was also considered exclusion criteria. Patients with known alterations of the coagulation system, as well as patients with severe systemic illness, were also excluded. The study design is illustrated in Figure 1.

The patients underwent platelet function evaluation by PFA-100 with CEPI cartridges and LTA within an average of 110 ± 8 hours after PCI: on the basis of PFA-100 analysis we identified 121 patients with CT <190 sec. 116 patients gave their approval and were then randomized between the standard combination of low dose aspirin (100 mg) and clopidogrel (75 mg) (group 0, G0, *n* = 58) or a tailored antiplatelet therapy (group 1, G1, *n* = 58), treated with a double dose of aspirin (200 mg) and clopidogrel (150 mg), for a 6-month follow-up period.

A cut-off value of CEPI-CT of 190 sec was chosen to define HPR because it has been shown to identify patients at high risk for recurrent acute coronary events [10].

Clopidogrel was given to all patients as a loading dose of 600 mg. Periprocedural GpIIb/IIIa receptor antagonist Abciximab was used in 11 patients (9%), 6 in G0, and 5 in G1 (*P* = 0.56).

### 2.2. Blood Collection and Platelet Function Analysis

Blood samples for analysis of platelet function were collected into evacuated tubes (Vacutainer, Becton Dickinson) containing 3.8% citrate. Platelet function was evaluated using the Platelet Function Analyzer-100 (PFA-100; Dade Behring), as previously described [11]. All measurements were done from 1 to 4 hours after blood sampling. The reference range in normal subjects was 76–184”. Coefficients of variation for duplicate analysis averaged 15% with a day-to-day variability that was around 10% for both cartridges.

For aggregometry analysis, platelet-rich plasma, obtained by centrifuging whole blood for 10 minutes at 200 g, was stimulated with 10 *μ*M ADP (Mascia Brunelli, Milan, Italy) and 1 mM AA (Sigma-Aldrich, Milan, Italy) and residual drug aggregation was assessed using a APACT 4 light transmittance aggregometer (Helena Laboratories, Milan, Italy). The 100% line was set using platelet-poor plasma and the 0 baseline established with platelet-rich plasma (adjusted from 180 × 10^9^/L up to 300 × 10^9^/L). Platelet aggregation (according to the Born's method) was evaluated considering the maximal percentage of platelet aggregation in response to stimuli. Coefficients of variation of ADP-LTA and AA-LTA were 6.8% and 5.8%, respectively.

The VWF antigen (Ag) levels were measured in plasma using an ELISA kit (GTI Diagnostics, Waukesha, WI, USA). Briefly, prediluted calibrators and diluted plasma samples were added to microwells, coated with monoclonal antibodies specific for VWFAg; bound VWFAg was assessed with biotinylated anti-VWF Ag monoclonal antibody and streptavidin-labelled HRP. Reportable results are given in U/dL [12]. A commercially available ADAMTS13 activity assay (ADAMTS-13 Activity Assay, ATS-13, GTI Diagnostics, Waukesha, WI, USA) was used to measure enzyme activity according to the manufacturer's directions. The principle of this assay is based on fluorescence resonance energy transfer (FRET) technology in which a fluorescent signal is detected when a synthetic substrate (FRETS-VWF73) is cleaved by ADAMTS-13 [13].

### 2.3. Clinical Follow-Up

Follow-up data were prospectively collected at our outpatients' clinic, according to a routine protocol. The follow-up visits were conducted by cardiologists not involved in the study. The first clinical, instrumental, and laboratory evaluation was obtained 30 days after patients' discharge. Patients follow-up was completed by a clinical evaluation 6 months after-PCI.

Major acute coronary events or MACE were defined as the composite of death, recurrent acute coronary syndrome, and a thrombolysis in myocardial infarction (TIMI) flow 0 or 1 by angiography at the site of stent implantation (in-stent thrombosis) and readmissions for target lesion revascularization. Stent thrombosis was classified as acute (0 to 24 hours), subacute (>1 day and <30 days), and late (>30 days) in accordance with the Academic Research Consortium definition [14].

Major bleedings were defined as intracranial bleeding or clinically overt bleeding associated with a decrease in hemoglobin of 5 g/dL, according to the TIMI criteria. Minor bleedings were also defined according to TIMI criteria [15].

### 2.4. Statistics

Continuous variables are expressed as means ± SD and categorical data as percentages. Chi-square tests or Fisher's exact tests were used for qualitative variables, and Student's unpaired *t*-test was used for continuous variables. ANOVA analysis was used for intragroups comparisons at *T*0-*T*1-*T*2. Multivariate logistic regression was performed to identify independent correlates of CEPI-CT. After univariate analysis, all variables with *P* values < 0.20 were introduced in the multivariate analysis. The variables entered in the HPR multivariate model were VWF, ADAMTS-13, fibrinogen, fasting glycemia, systemic hypertension, dyslipidemia, diabetes, and proton pump inhibitors use.

Correlations between tests were assessed using Pearson's test or Spearman's test (when the distribution was not normal). All *P* values are 2 sided, and *P* values < 0.05 were considered significant.

Statistical analysis was performed with Stat-View software version 5.0.1 (SAS Inc., Cary, NC, USA).

## 3. Results

The control and the tailored groups had similar demographic, clinical, and hemodynamic characteristics, including the time-to-balloon and the rate of Abciximab infusion pre-PCI (Table 1). The prevalence of cardiovascular risk factors for thrombosis was similar, including diabetes, basal level of creatinine, left ventricular ejection fraction, and stent length/patient (*P* = 0.81, 0.74, 0.86, 0.73, resp.).

The door-to-balloon time was 100 ± 18 minutes in the overall population, without significant intergroup difference (Table 1).

**Figure 2:**
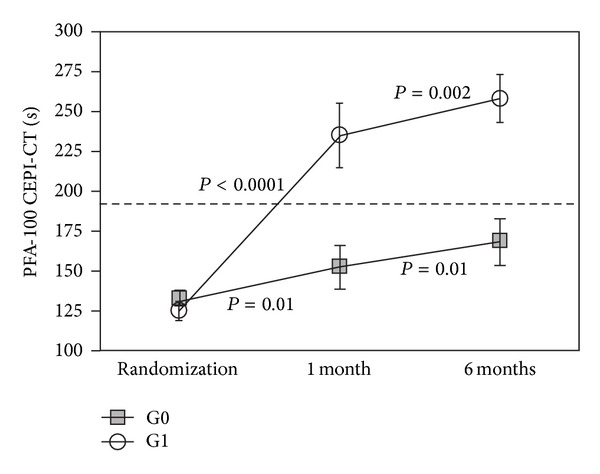
Ischemic and bleeding events during 6-month follow-up in G0 and G1.

A total of 7 major coronary events were recorded during the 6-month follow-up, including 1 cardiovascular death after 21 days (G0), 2 subacute in-stent thrombosis (both in G0), 1 late in-stent thrombosis (G1), and 3 target lesion revascularizations (2 in G1, 1 in G0). We observed a nonsignificantly lower rate of events in the tailored group (5.2%), compared with the control group (6.9%). We recorded also 2 minor bleedings, both in G1 (Figure 2).

**Figure 3:**
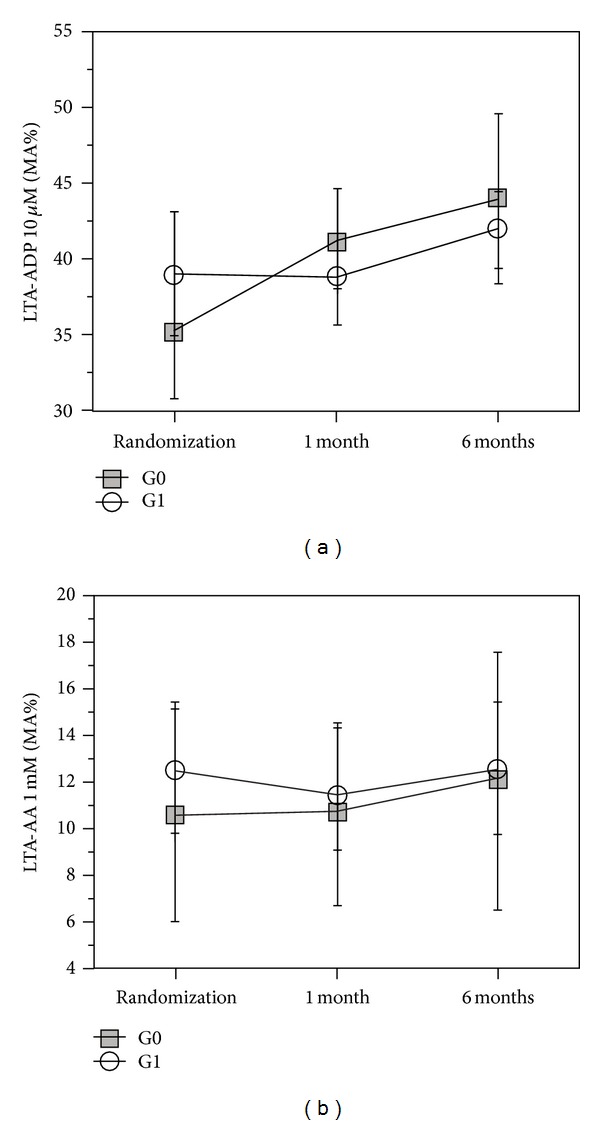
Results of CEPI-CT in G0 and G1 at 5, 30, and 180 days following PCI.

**Figure 4:**
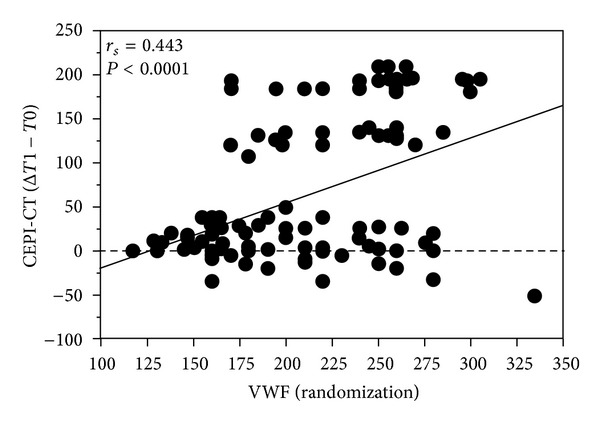
Results of LTA with arachidonic acid and ADP G0 and G1 at 5, 30, and 180 days following PCI.

**Figure 5:**
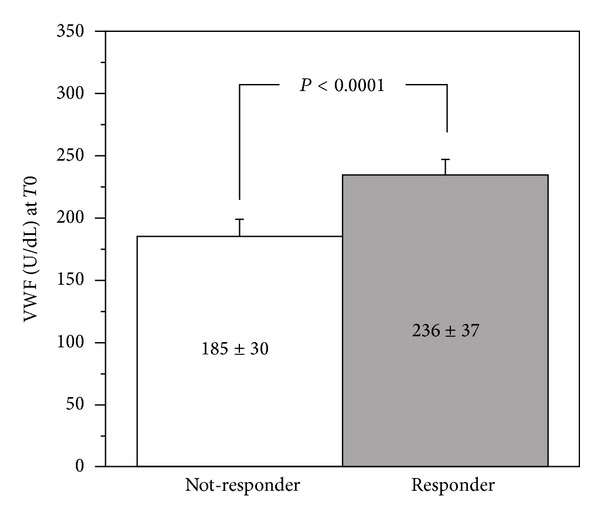
Correlation of delta of CEPI-CT (*T*1−*T*0) to basal levels of VWF and ADAMTS-13.

At Time 0, G0 and G1 were comparable also for platelet function tests results, VWF and ADAMTS-13 levels (Table 2). At Times 1 and 2, we observed a significantly higher CEPI-CT in G1 as compared to G0 (*P* < 0.001): 37 of 58 patients (63%) in G1 had a significant response to CEPI-CT at Time 1, whereas only 6 of 58 patients (9%) in G0 spontaneously converted to CEPI-CT responders (Figure 3). No difference was observed for either MA-AA or MA-ADP (Figure 3). Delta of CEPI-CT at *T*1−*T*0 was significantly related to VWF (*P* < 0.001) and to ADAMTS-13 (0.01) at Time 0 (Figure 4). In G1, responders versus nonresponders to CEPI-CT had significantly higher levels of VWF at Time 0 (Figure 5). Finally, in a multivariate model analysis, VWF resulted in significant predictor of CEPI-CT response (*P* = 0.009) (Table 3).

CEPI-CT values >200 sec had a significant negative predictive value (85%) in excluding VWF values >75° percentile (Table 4).

## 4. Discussion

This study provides the following original observations.

1. VWF predicts the response to a tailored strategy with double dose of aspirin and clopidogrel in reversing the HPR based on CEPI-CT values;
2. PFA-100 may be considered an indirect point-of-care method to rule out high levels of VWF antigen.

VWF binds exposed collagen to form a bridge between the site of vascular injury and platelets during the initiation of haemostasis. Due to its pivotal role in hemostatic plug formation, VWF may directly influence the likelihood of a thrombotic event, as suggested by the association of VWF levels with an increased risk of ischemic heart disease [16]. Plasma VWF is comprised of multimers held together by intermolecular disulfide bonds. Larger multimer are the most active at the site of vascular injury. VWF multimeric size is modulated by ADAMTS-13, which cleaves in the VWF A2 domain, thus reducing both the molecular weight and platelet-tethering function. The level of ADAMTS-13 in the blood may thus influence cardiovascular disease, especially in some population subgroups [17].

Previous studies have outlined the relationship of VWF level/activity and PFA-100 response, either in case of defective activity (Von Willebrand disease) or in case of hyperactivity (thrombotic disorders) [18].

Our data indicates that VWF basal levels are significantly correlated with the response of a tailored strategy, based on PFA-100 results, indicating that patients who benefit most from an intensified DAPT are those with the highest levels of VWF at the time of randomization (*T*0).

Moreover, HPR detected by PFA-100 does not correlate with the results of LTA-AA: none of the 116 patients with a definition of HPR at *T*0 exhibited a LTA-AA >20%, the cut-off value accepted to describe a condition of aspirin resistance [19].

In fact, CEPI-CT is a COX-1-independent test and poorly correlates with COX-1-specific tests, such as LTA-AA: the results of PFA-100 testing represent a complex interaction between drug effects, inflammatory reaction, and prothrombotic activity; conversely, laboratory tests solely exploring the AA-mediated pathway of platelet function, while being the most appropriate to detect the effect of aspirin on its pharmacologic target (COX-1), may fail to reveal the functional interactions between minimal residual thromboxane and additional stimuli or primers potentially leading to platelet aggregation [20]. In fact, HPR detected by CEPI-CT is largely correlated with biohumoral factors which substantially contribute to the process of arterial thrombosis in vivo, under flow conditions [21].

HPR, regardless of what might be detected, is a risk factor for recurrent ischemic events, particularly in-stent-thrombosis, following acute coronary syndromes [22]. In the last few years much attention has been focused on clopidogrel resistance as a major determinant factor in HPR [23–25]. However, so far, no clear evidence indicates that a tailored strategy based on the correction of clopidogrel resistance is clinically superior to a “one-size-fits-all” approach. Two of the largest randomized clinical trials of tailored versus standard therapy (ARCTIC and GRAVITAS studies) failed to demonstrate that, in clopidogrel resistant patients, defined by specific tests for ADP receptors-dependent pathways, a high-dose clopidogrel is superior [26, 27]; on the other hand, overall data indicate that a personalized strategy based on different methods, not strictly specific for P2Y12 pathway activation, may be successful, especially in high-risk patients [28].

Our data suggest that PFA-100 is an alternative approach to detect a state of HPR (CEPI-CT <190 sec) and its correlation with VWF is of interest in the context of novel antiplatelet targets under investigation, outside the P2Y12 receptor pathway, such as VWF inhibitors or TRAP receptor antagonists [29, 30]. The tailored strategy that we used (a double dose of aspirin and clopidogrel maintained for 6 months) was chosen since the proven effectiveness of a high dose of clopidogrel in the subset of high risk patients admitted for acute coronary syndromes (ACS) and treated by primary PCI in the CURRENT-OASIS-7 trial [31].

Few studies have assessed the antiplatelet effect of a 150 mg daily MD of clopidogrel, and at least 1 study that involved patients with STEMI showed that doubling the MD of clopidogrel (to 150 mg/d) during 30 days increased the degree of platelet inhibition and decreased the rate of hyporesponders to the drug [32]. There is no information available on the long-term benefit of 150 versus 75 mg of clopidogrel. In the analysis of TRITON and PLATO, there was an exact same additional 20% relative reduction of ischemic events during the MD period (30 days to 12 or 15 months) for both trials for ticagrelor and for prasugrel, clearly suggesting that there is a benefit of a higher MD for a longer period than 7 days, at least on the ischemic side, and subsequently leading to the conclusion that the period of 7 days with 150 mg of clopidogrel in the CURRENT trial may have been too short [33]. A double dose of clopidogrel in our study did not result in a significant decrease of ADP-MA (*P* = 0.45) at *T*1 and *T2* (Figure 3): the possible explanation is that in our study the tailoring strategy was not guided by a P2Y12-specific test; at *T*0, only 11 out of 116 patients (9%) were clopidogrel resistant (5 in G0, 6 in G1), as defined by a 10 *μ*M ADP MA >50%; at *T*1, 8 patients were still clopidogrel resistant (5 in G0 and 3 in G1), without significant difference between groups (*P* = 0.27).

Regarding the optimal dose of aspirin in acute coronary syndromes, the Antithrombotic Trialists Collaboration meta-analysis has defined the lack of a relationship between increasing aspirin dose and improved efficacy, with lower doses favored by improved safety [34]. More recently, in CURRENT-OASIS 7 there was no significant difference in the primary outcome, with a nonsignificant reduction of ischemic events with the higher dose of aspirin [31]. Interestingly, compared with low-dose aspirin, higher aspirin doses up to 325 mg did not result in any significant difference in the primary outcome and in major bleeding. The relatively good safety of high-dose aspirin may be related to the short-term use of these higher doses compared with previous studies that reported safety problems with high doses for chronic use. Although in a low number of patients we also confirm that in a 6-month period a double dose of aspirin and clopidogrel did not significantly increase the rate of major bleedings (0 events), with a low increase in minor bleedings (only 2 cases in G1). This data seems to support the relative safety of a more aggressive DAPT approach, possibly for a short period after acute coronary syndromes.

**Figure 6:**
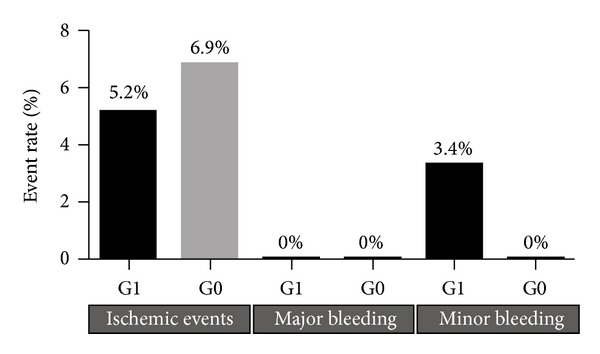
VWF levels in responders and nonresponders to tailored therapy (*T*1).

Moreover, patients with a persistent HPR at *T*1, as defined by a CEPI-CT <190 sec, showed lower levels of ADAMTS-13 (Figure 6), confirming previous data indicating the pathophysiological correlation between this matrix metalloprotease regulating the functional levels of VWF and the state of HPR [35].

## 5. Conclusions

VWF is a good predictor of response to a tailored DAPT strategy based on PFA-100 results. Moreover, the very good negative predictive value of a CEPI-CT >190 sec in ruling out VWF ≥75° percentile (Table 4) makes PFA-100 a reasonable and reproducible test in thrombotic risk stratification after acute coronary syndromes.

## 6. Limitations

This is a preliminary randomized study underpowered to detect clinically significant differences in MACE by a tailored DAPT strategy based on PFA-100 results. Thus, whether a tailored approach based on PFA-100 provides clinical advantages requires further investigations in larger randomized clinical trials.

## Figures and Tables

**Table 1:** Clinical, hemodynamic, and laboratory data of patients recruited.

	Group 0 (standard therapy) (*n* = 58)	Group 1 (tailored therapy) (*n* = 58)	*P* value
Male sex *n* (%)	41 (71)	38 (65)	0.51
Age*	59 ± 7	61 ± 11	0.33
BMI*	27 ± 2	27 ± 5	0.77
LVEF*	46 ± 4	45 ± 5	0.64
Current smoking, *n* (%)	16 (28)	16 (28)	0.98
LDL-Cholesterol*	129 ± 34	135 ± 14	0.51
Diabetes mellitus, *n* (%)	16 (28)	18 (31)	0.41
Hypertension, *n* (%)	31 (54)	35 (61)	0.27
Time to balloon, min*	112 ± 18	102 ± 18	0.42
Pre-PCI Abciximab, *n* (%)	6 (10)	5 (8)	0.59
Pre-PCI TIMI flow*	0.4 ± 0.2	0.5 ± 0.2	0.89
Post-PCI TIMI flow*	2.8 ± 0.3	2.9 ± 0.3	0.71
Number of diseased vessels*	1.6 ± 0.3	1.4 ± 0.4	0.86
Number of treated vessels*	1.2 ± 0.2	1.1 ± 0.2	0.78
Number of balloon/patient	2.8 ± 0.4	2.6 ± 0.4	0.43
Number of stents/patient*	1.4 ± 0.3	1.3 ± 0.3	0.65
Number of DES, *n* (%)	29 (50)	27 (48)	0.63
Stent length/patient, mm*	28 ± 7	26 ± 7	0.73
Time between PCI and PFA-100, h*	120 ± 10	120 ± 8	0.86
Hemoglobin, g/dL*	13.8 ± 3.2	13.1 ± 3.4	0.84
Leukocytes, g/L*	7.8 ± 1.8	7.2 ± 1.6	0.67
Platelets, 10^3^ g/L*	220 ± 53	241 ± 53	0.51
High sensitivity RCP, mg/dL	1.36 ± 0.8	1.22 ± 1.1	0.64
Fibrinogen, g/L*	3.2 ± 1.0	3.5 ± 0.9	0.18
Creatinine, mg/dL*	1.2 ± 0.3	1.1 ± 0.3	0.64
Cytochrome P450 metabolized drugs, *n* (%)**	58 (100)	57 (98)	0.97

BMI: body mass index; CAD: coronary artery disease; TIMI: thrombolysis in myocardial infarction; DES: drug eluting stents; LVEF: left ventricular ejection fraction; PCI: percutaneous coronary intervention; PFA: platelet function analyzer. *Mean ± standard deviation; **calcium channels antagonists, high dose statins.

**Table 2:** Platelet function tests results; VWF, and ADAMTS-13 levels.

	Group 0	Group 1	*P*
CEPI-CT (sec)			
T0	133 ± 24	125 ± 30	ns
T1	153 ± 43	235 ± 67	<0.01
T2	168 ± 46	258 ± 57	<0.01
AA-MA (%)			
T0	10.5 ± 7.3	12.4 ± 6.2	ns
T1	10.6 ± 11.2	11.6 ± 10.0	ns
T2	12.0 ± 16.3	12.5 ± 10.7	ns
ADP-MA (%)			
T0	35.3 ± 17.3	39.5 ± 16.7	ns
T1	41.5 ± 14.3	38.5 ± 16.7	ns
T2	44.3 ± 21.6	42.9 ± 10.3	ns
VWF antigen (U/dL)			
T0	207 ± 54	219 ± 42	ns
T1	193 ± 52	168 ± 37	<0.05
T2	195 ± 12	174 ± 44	<0.05
ADAMT S-13 activity (%)			
T0	81 ± 26	75 ± 23	ns
T1	80 ± 22	96 ± 22	<0.05
T2	85 ± 42	99 ± 37	<0.05

**Table 3:** Multivariate logistic regression for association with HPR (CEPI-CT < 190 sec) at *T*1.

Variable	Univariate analysis: *F* value/Chi square (*P* value)	Multivariate analysis: Chi square (*P* value)
Gender	1.068 (0.30)	—
Age	0.804 (0.37)	—
BMI	0.175 (0.67)	—
LVEF	0.766 (0.46)	—
Current smoking	0.589 (0.44)	—
LDL-Cholesterol > 130 mg/dL	3.102 (0.08)	—
Diabetes mellitus	3.363 (0.05)	9.151 (<0.001)
Hypertension	9.496 (0.001)	—
Number of diseased vessels > 1	1.403 (0.27)	—
Number of treated vessels > 1	1.117 (0.31)	—
PPI* use	3.602 (0.06)	—
Platelets	1.833 (0.18)	—
High sensitivity RCP	1.221 (0.34)	—
Fibrinogen	11.347 (0.001)	—
Creatininine	0.769 (0.47)	—
VWF	36.115 (<0.001)	6.873 (0.001)
ADAMTS-13	9.078 (0.003)	—

BMI: body mass index; LVEF: left ventricular ejection fraction; *Proton Pump Inhibitors.

**Table 4:** Negative predictive value of CEPI-CT > 190 sec for VWF > 75° percentile.

	CEPI-CT < 190 sec	CEPI-CT > 190 sec
VWF < 75°	46	38
VWF ≥ 75°	25	7

Negative predictive value: 38/45 = 84.5%.
